# 3D Reconstruction of Fishes Using Coded Structured Light

**DOI:** 10.3390/jimaging9090189

**Published:** 2023-09-18

**Authors:** Christos Veinidis, Fotis Arnaoutoglou, Dimitrios Syvridis

**Affiliations:** 1 Optical Communications Laboratory, Department of Informatics and Telecommunications, University of Athens, 15784 Athens, Greece; dsyvridi@di.uoa.gr; 2 “Athena” Research and Innovation Centre, Xanthi’s Division, 67100 Xanthi, Greece; fotarny@ceti.gr

**Keywords:** computer vision, 3D reconstruction, structured light, binary pattern

## Abstract

3D reconstruction of fishes provides the capability of extracting geometric measurements, which are valuable in the field of Aquaculture. In this paper, a novel method for 3D reconstruction of fishes using the Coded Structured Light technique is presented. In this framework, a binary image, called pattern, consisting of white geometric shapes, namely symbols, on a black background is projected onto the surface of a number of fishes, which belong to different species. A camera captures the resulting images, and the various symbols in these images are decoded to uniquely identify them on the pattern. For this purpose, a number of steps, such as the binarization of the images captured by the camera, symbol classification, and the correction of misclassifications, are realized. The proposed methodology for 3D reconstructions is adapted to the specific geometric and morphological characteristics of the considered fishes with fusiform body shape, something which is implemented for the first time. Using the centroids of the symbols as feature points, the symbol correspondences immediately result in point correspondences between the pattern and the images captured by the camera. These pairs of corresponding points are exploited for the final 3D reconstructions of the fishes. The extracted 3D reconstructions provide all the geometric information which is related to the real fishes. The experimentation demonstrates the high efficiency of the techniques adopted in each step of the proposed methodology. As a result, the final 3D reconstructions provide sufficiently accurate approximations of the real fishes.

## 1. Introduction

Three-dimensional (3D) imaging or 3D reconstruction is the process of determining the geometric structure of a scene based on a set of 2D images [1]. Generally, 3D reconstruction techniques can be divided into two categories: passive and active. In passive techniques, the optical components of the system simply record the scene without any additional emission of light. For instance, a stereo vision camera system consists of two cameras that capture the same scene. Similar to how the human vision system works, the desired 3D information in a stereo vision camera system is obtained by identifying the corresponding points between pairs of captured images. Active 3D reconstruction techniques, on the other hand, utilize at least one component that emits light onto the scene. One well-known and cost-effective technique for active 3D reconstruction is Coded Structured Light (CSL). In its simplest version, the CSL method utilizes a camera and a light source, which projects a pattern onto the scene. This pattern is a predefined image where each point is uniquely encoded. Consequently, extracting the corresponding points between the pattern and the image captured by the camera becomes a simpler and more accurate process. The final 3D reconstruction is achieved by utilizing these pairs of corresponding points and applying the triangulation technique.

Depending on the nature of the emitted pattern, various CSL techniques, such as time-multiplexing, direct coding, and spatial neighborhood, have been proposed in the literature [2]. In time-multiplexing techniques, multiple patterns are projected onto the scene of interest, with each point in the scene encoded by a unique code [3,4,5,6]. While these methods generally yield high-performance 3D reconstructions, they are not suitable for capturing moving objects. Direct coding techniques involve assigning a unique code, such as a color or grayscale value, to each point in the projected pattern [7,8]. However, these methods require a large number of codes, and the resulting 3D reconstructions are sensitive to noise. Spatial neighborhood methods enable the determination of each point in the pattern by decoding a group of neighboring symbols, referred to as a codeword [9,10,11,12,13]. Each codeword consists of a predefined number of symbols and appears only once in the pattern. These methods are well suited for the 3D reconstruction of dynamic scenes as the projected image is unique (one-shot methods).

The 3D reconstruction of an object provides all the information that characterizes its geometry in space. In the past, several works have utilized computer vision (CV) and/or Machine Learning (ML) techniques to estimate geometric features which are useful to the domain of Aquaculture. In [14], three Regional Convolutional Neural Networks (R-CNNs) ar trained in order to estimate the total length of European sea basses, using their public images. In [15], a system of two cameras acquires synchronized images, which are utilized to extract the keypoints of fishes, through the training of a CNN. These keypoints are exploited to estimate the length of Gilthead seabreams and European seabasses. In [16], the stereo images produced by an underwater stereo vision system are utilized to develop a keypoints R-CNN-based approach, in order to measure the lengths of five kinds of common freshwater fishes. In [17], two synchronized webcams constitute an automated and low-cost vision system, which is utilized to record two free-swimming adult zebrafishes. Using conventional methods, the corresponding images are processed to extract the binary masks of the recorded fishes. Using the calibration parameters of the system of the two cameras, the length of zebrafishes is estimated. In [18], a U-Net is trained after data augmentation to estimate the body length, the body width, and the body area of oval squids. In [19], a morphological image-processing algorithm is developed for the total length, body width, height, and weight of straightened flatfishes. In [20], several regressors based on CNNs are constructed to predict the length, the weight, and the circumference of various fish species. The total length of the fishes is determined using epipolar geometry, thanks to the calibration of the stereo system. In [21], the snout-to-fork length of fishes contained in complex images is presented. A CNN is developed to detect the fish head and the tail fork of the fishes, whereas snout and fork points are determined using image-processing techniques. In [22], CV techniques are used to determine the fish contained in a pair of images captured using a stereovision camera set. A comprehensive study of CV and ML techniques used for measuring the geometric characteristics of fishes, i.e., fish counting, fish recognition, and fish detection, among other applications is presented in [23].

In this paper, the problem of the 3D reconstruction of fishes with a fusiform body shape located outside of water is addressed using, for the first time, the CSL method. A new spatial neighborhood method, which utilizes a binary pattern emitted by the projector, is proposed. This pattern is constructed by placing the geometric symbols that form the various codewords as white symbols on a black background, enabling the decoding of symbols in the image captured by the camera, regardless of the color of the fishes onto which the pattern falls.

The image captured by the camera, which is a colored one, is converted to binary. The binarized image consists of a set of symbols that are transformed versions of the pattern’s symbols. These symbols are classified into one of the symbol classes initially used for forming the pattern. These classes were originally selected to have high separability among them, which is useful for classifying the symbols in the image that arises after the binarization of the camera-captured image.

One prerequisite for the success of any method using CSL is the local smoothness of the surface of the 3D object to be reconstructed. If this condition is satisfied within the extent of a symbol, the various symbols in the binarized version of the camera-captured image result from a linear transformation of the symbols as they appear in the pattern. This transformation is the projective transformation. In our case, where the 3D reconstruction of fishes with fusiform body shape is performed and their surfaces are continuous with relatively small curvature, it is demonstrated that considering only the fundamental geometric transformations is sufficient for the classification of the symbols in the images captured by the camera, with high accuracy.

After performing the classification of each symbol in the camera-captured images independently, the extraction of all submatrices of dimensions 3×3 (in total, consisting of nine symbols) takes place. These submatrices are called subpatterns. A property of the proposed pattern is that it is constructed in such a way that each subpattern is at a minimum Hamming distance of three from each other subpattern. This means that in each subpattern, there are at least three positions where a different symbol exists compared to the symbol present in the corresponding position of every other subpattern. This particular property of the pattern allows for the correction of an error in each subpattern [24], or more precisely, in the 1D version of each subpattern called a codeword. The codewords extracted from the images captured by the camera are compared to the known codewords of the projected pattern, and any codewords that do not differ in any symbol from a codeword of the pattern are considered successfully decoded. The same applies if a codeword of the images captured by the camera is found to differ in one position from a codeword of the pattern, utilizing the pattern’s property regarding error correction per codeword. By specifying these codewords, the nine symbols that constitute them are uniquely determined in the projected pattern. Finally, by using the centroids of the symbols as feature points from each of them, the desired correspondences of specific point pairs in the pattern and the images captured by the camera are unambiguously determined. After extracting the corresponding points, the 3D coordinates of various points of the fishes are evaluated by applying the triangulation method [25]. In this method, the 3D coordinates are calculated by utilizing the extracted pairs of corresponding points and known geometric measurements, such as the distance between the projector and the camera, as well as the focal lengths of both the camera and the projector.

The remainder of this paper is organized as follows: In Section 2, the characteristics of the proposed pattern are described, and the individual steps of the methodology for finding corresponding points between the template and the captured camera images are analyzed. In Section 3, the results obtained by applying the proposed methodology to four types of fishes are presented. Section 4 provides an extensive discussion of the results, while Section 5 summarizes the main findings.

## 2. Materials and Methods

**Figure 1:**
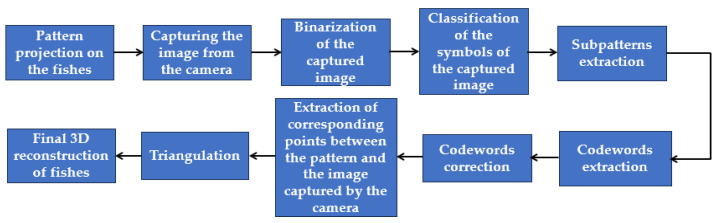
The general pipeline of a typical methodology which utilizes a spatial neighborhood method technique.

In Figure 1, the overall pipeline of the 3D reconstruction process using a typical spatial neighborhood method is illustrated. Also, in the next paragraph, several state-of-the-art papers that employ spatial neighborhood methods are discussed.

In [9], a binary pattern is utilized, consisting of subpatterns formed by 6 geometric symbols. Otsu’s method [26] is employed for the binarization of the image captured by the camera, while geometric criteria based on each symbol are used for symbol classification, as depicted in the camera-captured image. The orientation of dashes—one of the 6 different types of geometric shapes used—is employed for extracting codewords. Finally, correction is performed up to one error per codeword, based on the corresponding correction capabilities of the designed pattern. In [10], the binary pattern consists of 3 geometric symbols, exhibits center symmetry, and offers correction capabilities up to one error per codeword. Symbol classification, once again, relies on geometric properties characteristic of each symbol. In [11], a general pattern encoding method is proposed, while the ultimately used pattern is binary and composed of subpatterns formed by 3 geometric symbols. Subsequently, the contours of the geometric symbols in the image captured by the camera are extracted, and their classification is based on the training of a deep Lenet-5 CNN network. In [12], another binary pattern is proposed, formed by 8 symbols. The classification of the basic symbols in the camera-captured image relies on the classification of the corresponding skeletons of the symbols by a Deep CNN based on LeNet. In [13], the process of constructing patterns that include all possible subpatterns is described, without specifying directions for the decoding process, while error correction capabilities are not expected to be part of the pattern. The methodology proposed in our paper includes a fully unsupervised technique for symbol classification, eliminating the need for a separate stage of data collection and training, as is the case in works where classification is achieved through neural networks. Compared to other fully unsupervised techniques, improvements have been made in various individual steps of the overall 3D reconstruction algorithm.

In the following paragraphs, our entire methodology used for extracting the desired 3D reconstructions of fishes is described. Initially, the proposed pattern is described along with relevant details. Then, the proposed methodology for extracting corresponding points between the pattern emitted by the projector and the images captured by the camera is presented.

### 2.1. Coded Structured Light (CSL) Pattern

**Figure 2:**
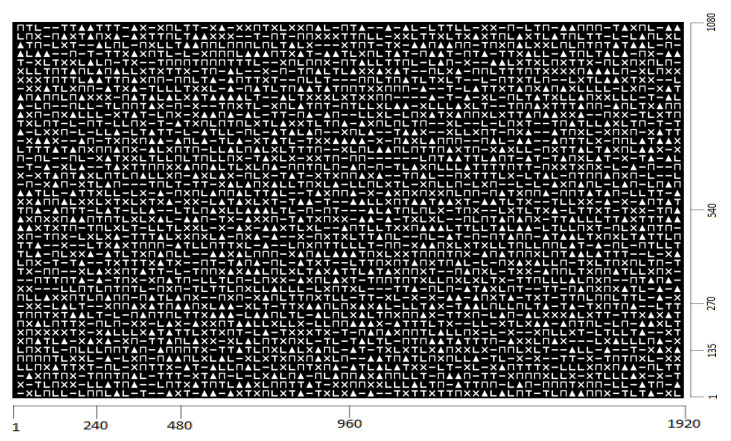
The pattern emitted by the projector.

The pattern projected on the fishes is the binary image shown in Figure 2.

**Figure 3:**
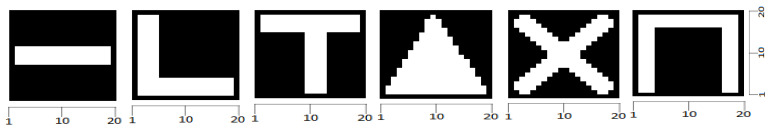
The 6 symbols used to create the pattern.

The pattern has dimensions of 1920×1080 pixels and consists of 76×43 symbols. To create this pattern, 6 symbols are used, which are shown in Figure 3. These symbols are a rectangle, an ‘L’, a ‘T’, a triangle, an ‘X’ and a ‘Π’.

**Figure 4:**
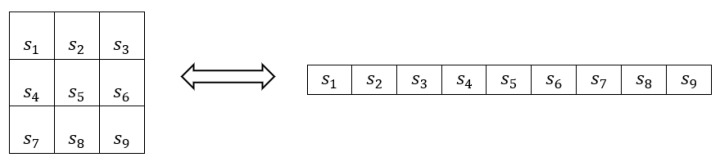
The sequence of symbol recording for transforming a subpattern into a codeword and vice versa, s_1_–s_9_ represent the symbols of a subpattern.

Each symbol in Figure 3 consists of 20×20 pixels. During the formation of the pattern, there is a 5-pixel gap between consecutive symbols, both horizontally and vertically. From each submatrix of the consecutive symbols with dimensions 3×3, called a subpattern, a codeword can be generated by adopting a specific sequence of symbols recording to transform the 2D arrangement of the subpattern into the 1D arrangement of the codeword. In our case, this sequence is illustrated in Figure 4.

The pattern is designed using the encoding algorithm presented in [27], in such a way that each 3×3 subpattern has a minimum Hamming distance of at least 3 from any other subpattern of the same dimensions. In other words, for the Hamming distance $HD\left(SP_{i},SP_{j}\right)$ between a subpattern $SP_{i}$ and any other subpattern $SP_{j}$ in the pattern, with i≠j, the following holds true:

(1) $$HD\left(SP_{i},SP_{j}\right)=\overset{3}{\underset{k_{1}=1}{\sum }}\overset{3}{\underset{k_{2}=1}{\sum }}SP_{i}\left(k_{1},k_{2}\right)⊕SP_{j}\left(k_{1},k_{2}\right)\geq 3,\;\mathrm{for}\;\mathrm{each}\;i\neq j\;$$

where:

(2) $$SP_{i}\left(k_{1},k_{2}\right)⊕\;SP_{j}\left(k_{1},k_{2}\right)=\left\{\begin{array}{c} 0,\;if\;SP_{i}\left(k_{1},k_{2}\right)\neq SP_{j}\left(k_{1},k_{2}\right)\\ 1,\;if\;SP_{i}\left(k_{1},k_{2}\right)=SP_{j}\left(k_{1},k_{2}\right) \end{array}\right\}\;$$

while $SP_{i}\left(k_{1},k_{2}\right),SP_{j}\left(k_{1},k_{2}\right)$ refers to the symbol at position $\left(k_{1},k_{2}\right),\;1\leq k_{1},k_{2}\leq 3$, in the subpatterns $SP_{i},\;SP_{j}$, respectively. Obviously, each codeword also has a minimum Hamming distance of 3 from any other codeword in the pattern. This property of the pattern enables the ability to correct one error per codeword using the Hamming code, which is further utilized as described in the subsequent Sections.

### 2.2. Binarization of the Captured Images

**Figure 5:**
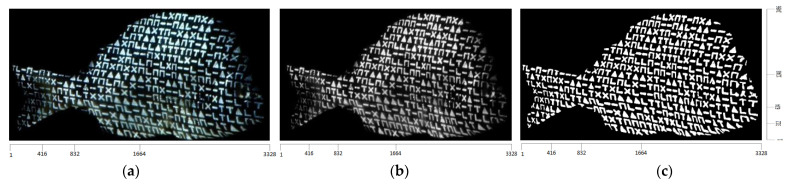
(**a**) The RGB image captured by the camera, (**b**) the corresponding grayscale image, (**c**) the resulting binary image after Sauvola’s method.

The pattern shown in Figure 2 is emitted by the projector and illuminates the surface of the fishes used for the experiments, while the camera captures these surfaces as RGB images. In Figure 5a,b, such an RGB image as well as the corresponding grayscale image are, respectively, shown. This grayscale image is transformed to binary using Sauvola’s method [28]. This technique, unlike cases of global binarization algorithms such as Otsu’s method, where a unique threshold is determined for the entire image, different thresholds are extracted for different regions of the image. Such a technique has the advantage of not being affected by possible illumination variations in the image. In Figure 5c, the binarized version of the image Figure 5b is shown after applying Sauvola’s method and rejecting the Connected Components (CCs) consisting of fewer than 50 pixels, which are considered as noise.

### 2.3. Classification of Symbols of the Captured Images

Let $I_{c}$ be the image obtained after binarizing the image captured by the camera, using Sauvola’s method and rejecting the CCs consisting of fewer than 50 pixels, as described in the previous Section. In general, if the assumption of the local smoothness of the surfaces of 3D objects is satisfied, the pattern undergoes a projective transformation after its incidence on the 3D object. This results in a transformed version of the pattern’s symbols compared to their form in the projected pattern. However, for the specific case of the fishes with fusiform body shape, where the curvature of their bodies is relatively small, it is sufficient to consider the fundamental transformations of rotation and scaling in order to achieve an effective classification of the image symbols captured by the camera. Let $s_{1}$ be a symbol belonging to $I_{c}$. The proposed algorithm for classifying $s_{1}$ consists of the following steps:

- Initially, the Bounding Box (BB) of $s_{1}$, which is the minimum area rectangle, with vertical and horizontal sides that encloses the entire symbol, is determined.
- Subsequently, each of the 6 valid symbols as they appear in the original pattern is generated. In total, 45 different variations are generated for each of these 6 symbols. These variations are obtained by considering all rotations of each symbol by an angle $\theta \in \left[-22,22\right]$, where consecutive rotations differ by one degree.
- For each of the 45 rotated variations of each symbol, the corresponding ΒΒ is extracted and the lengths of its sides are determined. Then, the ratio of the height as well as the ratio of the width of this BB to the corresponding dimensions of the BB of $s_{1}$ are evaluated. Each dimension of the 45 rotated variations of each symbol is elongated or shrunken accordingly, based on the ratio of their respective dimensions of the BB. This way, the different rotated variations of the symbols, along with $s_{1}$, which will be compared later, are brought to the same scale.
- As a measure of dissimilarity between each of the 45 rotated variations of each symbol and $s_{1}$, a normalized version of the Sum of Absolute Differences (SAD) is used. Specifically, the SAD is calculated between the symbols being compared, normalized by the maximum number of white pixels in both symbols. Formally, if X and Y are the binary images that contain only the corresponding symbols, then the measure of their dissimilarity is as follows:

(3) $$SAD\left(X,Y\right)=\frac{\sum_{i=1}^{N_{1}}\sum_{j=1}^{N_{2}}\left|X\left(i,j\right)-Y\left(i,j\right)\right|}{\underset{\;}{\max}\left\{\sum_{i=1}^{N_{1}}\sum_{j=1}^{N_{2}}X\left(i,j\right),\sum_{i=1}^{N_{1}}\sum_{j=1}^{N_{2}}Y\left(i,j\right)\right\}}\;$$

where $N_{1}\times N_{2}$ are the dimensions of the images X and Y, respectively.

- The final measure of dissimilarity between a transformed symbol and $s_{1}$ is defined as the minimum dissimilarity among the 45 individual dissimilarities, expressed by Equation (3).
- Finally, $s_{1}$ is classified into the symbol class with the minimum dissimilarity among the six corresponding values computed in the previous step of the classification algorithm.

### 2.4. Extraction of Subpatterns and Codewords

A unique subpattern of dimensions 3×3 symbols is extracted, with the central symbol being the symbol $s_{1}$ itself. Initially, the eight nearest symbols to $s_{1}$ are considered. In most cases, these symbols form the subpattern with the $s_{1}$ as central symbol. If this is not the case, then the specific subpattern is not considered valid, based on the criteria mentioned below.

In order to fully determine a subpattern, the arrangement of its symbols, following the same order as defined in the original pattern, must be specified. For this purpose, a process of successive determinations of the closest neighboring symbols belonging to the subpattern is implemented, as described below:

- Initially, the nearest symbol of $s_{1}$ among the remaining eight symbols of the subpattern is determined. Let this symbol be denoted as $s_{2}$.
- Excluding $s_{1}$, the nearest symbol to $s_{2}$ is determined among the remaining seven symbols of the subpattern.
- The above process of successive exclusions of symbols that have already been visited and then determining the nearest symbol each time is repeated until all symbols of the subpattern have been visited.

**Figure 6:**
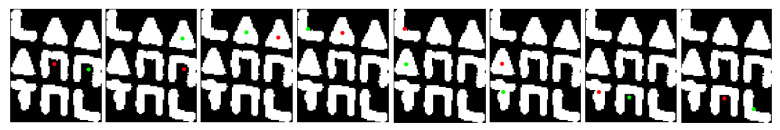
The successive steps for extracting the nearest symbol for each symbol of the subpattern. The symbol we visit in the current iteration is marked in red, and the symbol that represents its closest neighboring symbol within the subpattern is marked in green.

The successive steps of the aforementioned procedure are shown in Figure 6.

**Figure 7:**
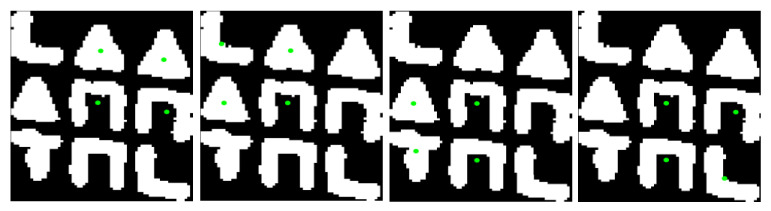
The four 2×2 grids of neighboring symbols which compose the 3×3 subpattern. The four symbols of each 2×2 grid are marked by green dots.

As shown in this Figure 6, in each step of the algorithm, a directly neighboring symbol is determined from the symbol last visited, in the vast majority of cases. Based on this assumption, it is feasible to determine the arrangement of symbols in the subpattern. The crucial observation is that each 3×3 subpattern can be generated by the horizontal and vertical concatenation of four overlapping 2×2 grids of neighboring symbols. These grids of symbols are illustrated in Figure 7.

**Figure 8:**
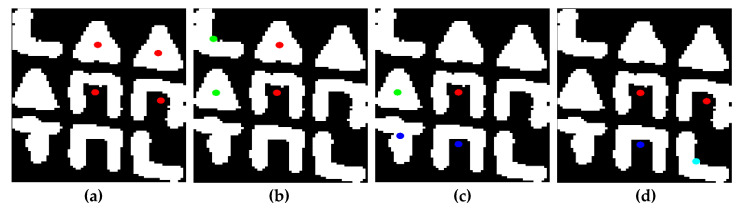
The successive steps for extracting the four 2×2 grids of symbols. In (**a**), the symbols that form the first 2×2 grid are marked in red, while in (**b**–**d**), symbols extracted for the first time in the second, third, and fourth steps of the corresponding algorithm, respectively, are marked in green, blue, and cyan color.

Following the steps of the above algorithm, which extracts the nearest neighbor of each symbol, and based on the aforementioned assumption regarding the succession of symbol visits, it follows that the four individual 2×2 grids of symbols that compose the entire codeword are determined after successive steps of symbol visits. Specifically, the first 2×2 grid of symbols is determined after three symbol visits, the second one after an additional two visits, the third one after two more visits, and the fourth one after the last visit, as shown in Figure 8.

In this Figure, starting from the central symbol, the one immediately to the right is determined as its closest neighboring symbol. After finding two additional closest neighboring symbols and following a counterclockwise direction, the first 2×2 grid of symbols is determined, as shown in Figure 8a. The two subsequent symbols that are determined, complete the second 2×2 grid of symbols, as shown in Figure 8b. The third one is determined by extracting two additional nearest neighboring symbols, as shown in Figure 8c. The last symbol belonging to the 3×3 subpattern is determined after a final step, as shown in Figure 8d.

The final extraction of the subpattern is performed by realizing a dual determination of the relative position, both among the 2×2 grids of symbols and among the symbols within each 2×2 grid of symbols. The corresponding algorithm is the same, where it is applied once considering the centroids of the four 2×2 grids of symbols as input, and another time considering the centroids of the four symbols that constitute each of the four 2×2 grids of symbols as inputs.

**Figure 9:**
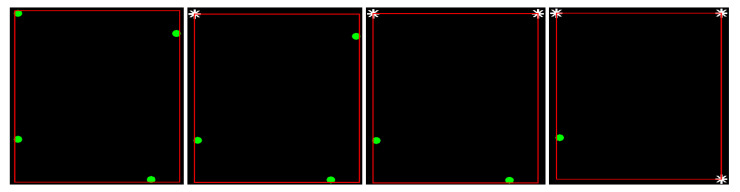
The successive steps for projecting a group of 4 points onto their BB vertices, with green dots representing the 4 points and white stars indicating the BB vertices that are not considered in subsequent algorithm steps.

The four input points in this algorithm are projected onto the four corners of their BBs, where the problem of finding the relative position of these corners is trivial. Specifically, the distance of each point from each of the four corners of their BBs is initially calculated. The pair of the given point and the BB corner, for which the corresponding minimum distance is obtained, indicates the corner of the BB onto which this point is projected. By excluding this point and corner from subsequent calculations, the pair of the input point and the BB corner are determined, which yields the minimum distance value among the remaining three points and corners of the BB. This process is repeated until all points are assigned to a corner of their BBs. This process is illustrated in Figure 9.

**Figure 10:**
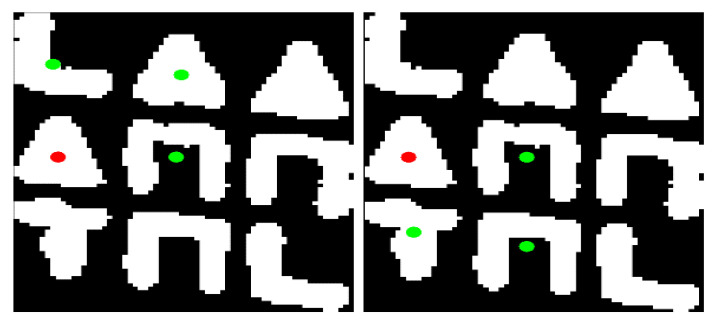
Two separate 2×2 grids of symbols with a common symbol, specifically a triangle, marked with a red dot. The remaining 3 symbols of each grid are marked with green dots. In the left grid, the triangle is the bottom-left symbol, while in the right grid, the triangle is the top-left symbol.

**Figure 11:**
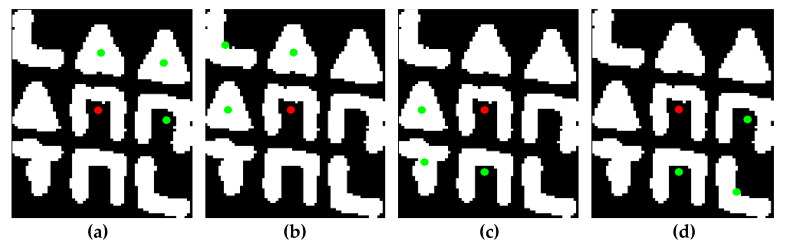
Four 2×2 grids of symbols with a common symbol, specifically a ‘Π’ symbol, marked with a red dot. The remaining 3 symbols of each grid are marked with green dots. In the various grids, this symbol occupies all four possible positions it can be in, specifically: bottom-left in (**a**), bottom-right in (**b**), top-right in (**c**), and top-left in (**d**).

After the above process, the relative position is extracted both among the grids of 2×2 symbols and among the symbols within each of the four grids of 2×2 symbols. As a result, it is feasible to uniquely determine the position of each symbol within the 3×3 subpattern. Specifically:

- The top-left symbol of the subpattern is determined as the top-left symbol of the top-left 2×2 grid of symbols.
- The top-right symbol of the subpattern is determined as the top-right symbol of the top-right 2×2 grid of symbols.
- The bottom-left symbol of the subpattern is determined as the bottom-left symbol of the bottom-left 2×2 grid of symbols.
- The bottom-right symbol of the subpattern is determined as the bottom-right symbol of the bottom-right 2×2 grid of symbols.
- Excluding the central symbol of the 3×3 subpattern, each of the middle symbols, i.e., those located between two other symbols either horizontally or vertically, can be determined in two ways. For example, the central-left symbol can be simultaneously determined as the bottom-left symbol of the top-left 2×2 grid of symbols and as the top-left symbol of the bottom-left 2×2 grid of symbols, as shown in Figure 10. This capability to have a dual determination of these symbols provides a means of validating the correctness of the 3×3 subpatterns. Specifically, if there is at least one middle symbol that does not satisfy the dual checks using the four previously mentioned dual determinations, then the particular codeword is not considered valid.
- The central symbol of the 3×3 subpattern is part of the four 2×2 grids of symbols, thus providing a quadruple check regarding its position in these configurations. Specifically, this symbol simultaneously serves as the bottom-right symbol of the top-left configuration, the bottom-left symbol of the top-right configuration, the top-right symbol of the bottom-left configuration, and the top-left symbol of the bottom-right configuration, as shown in Figure 11. If it is found that at least one of the four aforementioned checks does not hold true, then the corresponding codeword is rejected. In other words, if any inconsistency is detected in any of these four checks, the subpattern is considered invalid and is discarded.

### 2.5. Corresponding Points Extraction

After determining the valid subpatterns, they are transformed into codewords. For this purpose, the inverse transformation from the one shown in Figure 4 is used to convert the 2D symbol arrangements into 1D. Τhe Hamming distance between these codewords and the valid codewords of the pattern is calculated. Since the pattern has the capability to correct one error per codeword, initially, valid codewords with a Hamming distance equal to 0 or 1 from a valid codeword of the pattern are identified. These codewords are accepted as valid. In the case of codewords with a minimum Hamming distance of 1 from a valid codeword of the pattern, the correction of the unique misclassified symbol is extended to all the remaining codewords in which this symbol exists. In this way, the minimum Hamming distance of those codewords from the corresponding pattern codewords is reduced, which may allow the further correction of one or more of these codewords. The above iterative process of symbol correction continues until there is no possibility of further symbol corrections.

Following the process described above, it is feasible to achieve an unambiguous determination of correspondences between individual symbols of the pattern and symbols of the image $I_{c}$. By using the centroids as representative points for the symbols in both cases, it is possible to achieve a final explicit determination of pairs of corresponding points between the pattern and the images captured by the camera.

## 3. Results

**Figure 12:**
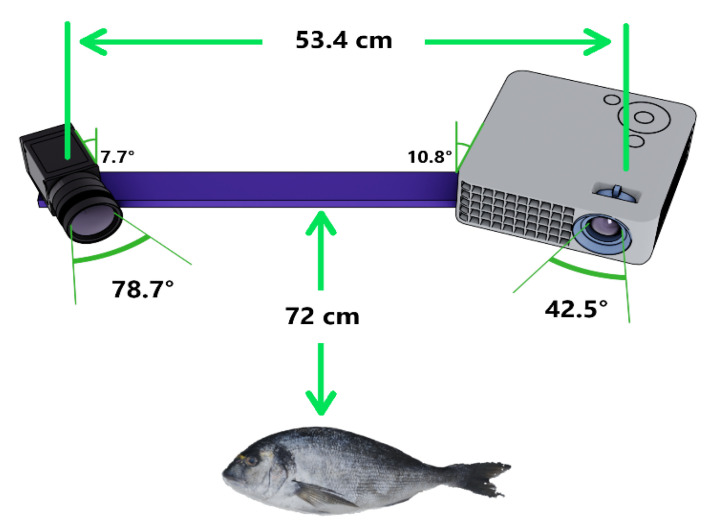
The projector–camera system. The distance between them, the respective Fields Of View, and the distance at which the fishes are positioned are also indicated.

The algorithm is implemented with MATLAB 2019a. The experimental setup is configured with an infocus IN138HD projector with a resolution of 1920×1080 pixels and a camera Panasonic Lumix DC-BGH1 with a resolution of 3328×2496 pixels. The projector uses a lens with an F-Stop of 2.43, a horizontal Field Of View of 42.5 degrees, and a contrast ratio of 28,500:1. The camera uses a lens with an F-Stop of 1.7 and a horizontal Field Of View of 78.7 degrees. The distance between the camera and projector is approximately 53.4 cm, and the fishes are placed at a distance of approximately 72 cm from them. In Figure 12, the imaging system is shown. This includes the light source (the projector), the camera, as well as the distances and the angles used in the triangulation stage, and the respective Fields Of View.

The signal-to-noise ratio (SNR, in dB) of the images captured by the camera for each of the four fish cases used in the experiments is summarized in Table 1.

**Figure 13:**
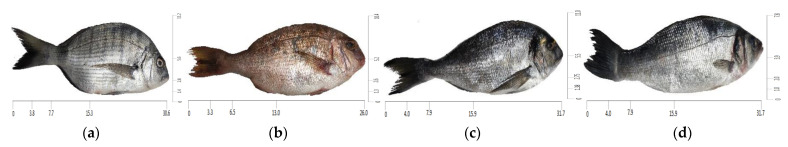
The fishes used for the experiments: (**a**) white seabream, (**b**) red seabream, (**c**) gilthead seabream, (**d**) European seabass.

Within the experimental framework, the 3D reconstruction of four fishes belonging to different species was conducted. Specifically, a white seabream (*Diplodus sargus*), a red seabream (*Pagrus major*), a gilthead seabream (*Sparus aurata*), and a European seabass (*Dicentrarchus labrax*) are used as reference objects for 3D reconstruction. This selection was made to encompass a number of variations in the characteristics of the fishes. For example, the red seabream has a different color (red) compared to the other three fishes, which primarily have gray shades. Additionally, the geometric characteristics of these fishes are distinct. The fishes used in these experiments are shown in Figure 13.

**Figure 14:**
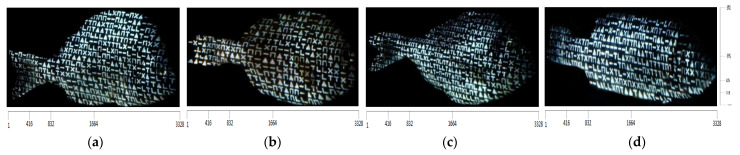
The images captured by the camera after the pattern falls onto the bodies of the fishes: (**a**) white seabream, (**b**) red seabream, (**c**) gilthead seabream, (**d**) European seabass.

**Figure 15:**
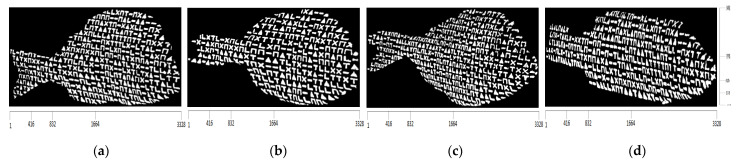
The resulting binarized images: (**a**) white seabream, (**b**) red seabream, (**c**) gilthead seabream, (**d**) European seabass.

After the incidence of structured light on the surfaces of the bodies of the four fishes, the images captured by the camera are shown in Figure 14 and the corresponding binary images are shown in Figure 15.

Due to the contrast observed at the various regions on the surface of the fish, as a result of the structured light applied by the projector, the binary representations of the corresponding images yield representations composed of distinct and discernible symbols. These symbols generate an artificial texture on the surface of the fishes’ body, which is used to facilitate the process of finding point correspondences between the projected pattern and the images captured by the camera. An important characteristic is that these results remain consistent regardless of the color of the fishes’ body surface, which is achieved by designing the pattern to be binary.

**Figure 16:**
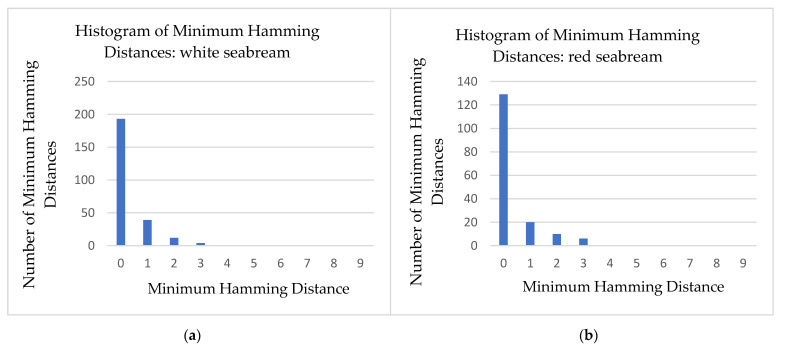

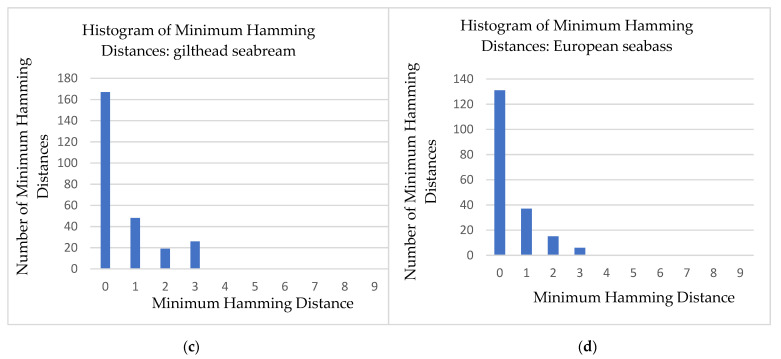
The histograms of the minimum Hamming distances between the codewords extracted from the images $I_{c}$ and the valid codewords of the pattern, as a function of the possible corresponding minimum Hamming distances: (**a**) white seabream, (**b**) red seabream, (**c**) gilthead seabream, (**d**) European seabass.

Each one of the binarized images consists of a set of CCs, which are considered valid symbols. These symbols are classified into one of the six acceptable symbol classes using the algorithm described in Section 2.3. Then, all possible 3×3 subpatterns of directly neighboring symbols are determined, based on the technique provided in Section 2.4. In addition, the subpatterns are transformed to 1D codewords, using the transformation of Figure 4. As a measure of the efficiency for both evaluating the proposed symbol classification algorithm and correctly extracting the codewords, the histogram of the minimum Hamming distances between these codewords and the codewords contained in the projected pattern, before the correction process described in Section 2.5, is presented in Figure 16. In addition, Table 2, Table 3, Table 4 and Table 5 summarize the final number of symbol pairs ultimately used for finding the final symbol correspondences between the projected pattern and the images captured by the camera.

**Figure 17:**
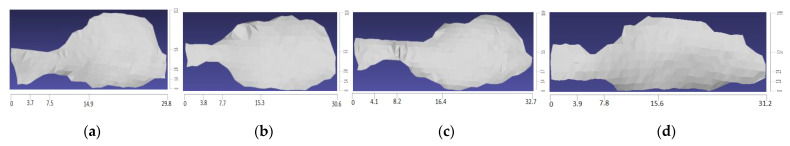
The final 3D reconstruction of (**a**) white seabream, (**b**) red seabream, (**c**) gilthead seabream, (**d**) European seabass.

Using the extracted symbol correspondences between the pattern and the images captured by the camera, it is feasible to determine the correspondences between points in both parts of the setup. Specifically, by using the centroids of the symbols as feature points, each pair of corresponding symbols can be directly converted into a pair of corresponding points. Using these point correspondences, the resulting 3D reconstructions, which are shown in Figure 17, are obtained.

**Figure 18:**
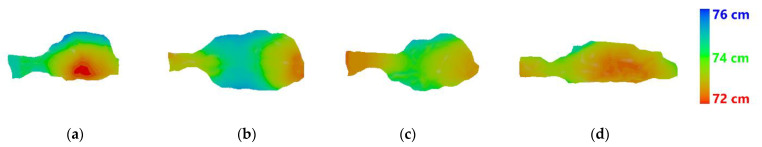
The depth maps corresponding to the fishes reconstructed in our paper: (**a**) white seabream, (**b**) red seabream, (**c**) gilthead seabream, (**d**) European seabass. On the right, a color bar is displayed, representing the color-depth mapping for various points in the 3D reconstructions.

The depth maps, which correspond to the 3D reconstructions of the fishes used in our experiments, are shown in Figure 18.

Given that the fishes are located at a distance of 72 cm from the projector–camera system, the depth values for the surfaces of the fishes range between 72 and 76 cm.

**Figure 19:**
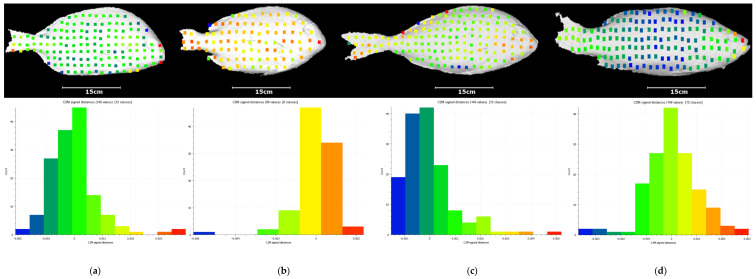
The Ground Truth (GT) models of the fishes reconstructed in our paper: (**a**) white seabream, (**b**) red seabream, (**c**) gilthead seabream, (**d**) European seabass, and the corresponding signed distances histogram between the points resulting from our 3D reconstructions and their corresponding nearest points in the reconstructions derived from the GT data. The colored points on the GT models represent the points resulting from our 3D reconstructions. The colors of these points correspond to the colors of the signed distances shown in the histograms above.

For assessment purposes, Ground Truth (GT) models of our fish subjects using a methodology called Structure From Motion (SFM) are obtained. By leveraging a commercial photogrammetry software and utilizing 100 images, each with a resolution of 16 megapixels, high-resolution and high-density models for each fish are generated. The resulting GT 3D models from SFM exhibit a median spatial resolution of approximately 0.6 mm. Figure 19 depicts the corresponding GT models. The colored points on the GT models represent the points resulting from our 3D reconstructions. The colors of these points correspond to the colors of the signed distances shown in the histograms below. Additionally, at the lower part of the same figure, the histograms depict the signed distances between the points resulting from the reconstructions of Figure 17 and their corresponding nearest points in the reconstructions derived from the GT data.

## 4. Discussion

**Figure 20:**
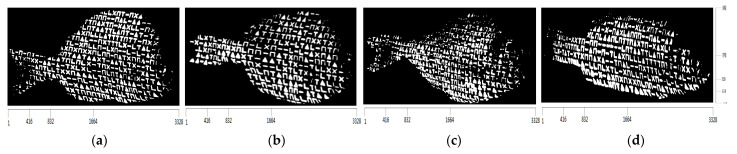
Binarization results using Otsu’s method for (**a**) white seabream, (**b**) red seabream, (**c**) gilthead seabream, (**d**) European seabass.

As shown in Figure 15, Sauvola’s adaptive binarization method is suitable for cases where images are unevenly illuminated. For comparison reasons, Figure 20 presents the corresponding binarization results using Otsu’s global technique.

It is evident that the binarized images resulting from Otsu’s method lead to images where symbols in some areas overlap while completely disappearing in certain others. This complicates subsequent algorithm steps since the number of the usable symbols is reduced, the classification of the symbols becomes less reliable, and the resulting valid codewords are fewer. On the other hand, when using Sauvola’s method, the symbols that cannot be utilized are mainly located at the boundaries of the fish bodies, where they are inevitably cut off and either discarded as noise in the algorithm’s further processing or often misclassified because a significant portion of them is lost. However, this limitation is inherent to the structured light in the 3D reconstruction technique and is not related to the specific methodology itself.

Figure 16 and Table 2, Table 3, Table 4 and Table 5 demonstrate that the method used for symbol classification and the algorithm for extracting subpatterns provide several codewords that can be utilized for the desired decoding of symbols. Specifically, it is evident that the overwhelming majority of extracted codewords have a minimum Hamming distance of 0 or 1 from one of the valid codewords extracted from the pattern shown in Figure 2. Given that even those codewords with a minimum Hamming distance of 1 from a pattern codeword contain a classification error that can be corrected, it can be inferred that the classification algorithm and codeword extraction process are sufficiently effective for the specific case of 3D reconstructions. Specifically, while in the general case, it is necessary to determine the parameters of the projective transformation applied to the original symbols, in order to generate the symbols in the image captured by the camera, in the case of the fishes used in our experiments, as well as many other fish species, it is largely sufficient to investigate only the fundamental geometric transformations of scaling and rotation. This is due to the specific characteristics of the fishes with a fusiform body shape, which exhibit relatively smooth variations in their curvature, particularly in areas of the fish body away from their edges, as well as the fact that the selected symbols in the pattern already have significantly large SAD among them. This characteristic largely persists in the symbols contained in the images captured by the camera. The normalized SAD matrix, given in Equation (3), between the symbols shown in Figure 3 is presented in Table 6.

From Table 2, Table 3, Table 4 and Table 5, it can be concluded that in all cases, a relatively small number of codewords is necessary to be decoded in order to obtain a significantly larger number of corresponding point pairs between the pattern and the images captured by the camera. This is due to the fact that decoding a codeword results in the decoding of nine symbols in our case, where each codeword consists of nine symbols. A symbol is considered fully decoded when it belongs to at least one decoded codeword.

**Figure 21:**
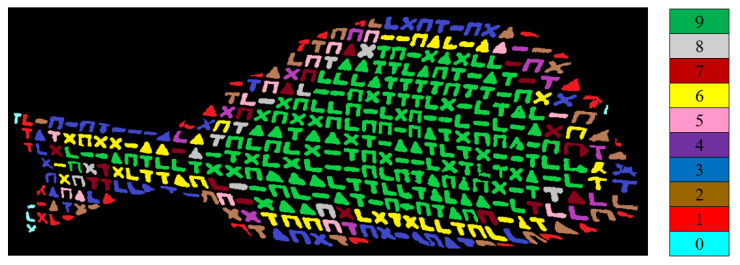
The number of codewords in which each symbol participates. The different colors indicate the corresponding number of each codeword, according to the column at the right of the image.

An additional reason why larger losses in decoded symbols are observed at the boundaries of the object for 3D reconstruction is related to the fact that those symbols participate in the formation of fewer codewords compared to a symbol located in a more central region of the object. To better understand this, in Figure 21, the image captured by the camera for white seabream is shown, and the number of codewords in which each symbol appears is indicated with different colors. As each codeword consists of nine elements, each symbol can participate in the formation of zero to nine codewords. This number is determined by the color with which each symbol is highlighted in the figure, according to the color table provided next to the figure.

It is evident that symbols surrounded more by other symbols, for example, those with green color, participate in the formation of a greater number of codewords. These symbols are more likely to be decoded correctly, as there are more chances for correctly decoding at least one corresponding codeword. On the other hand, symbols near the boundaries of the object, for example, those with blue color, are less likely to be decoded correctly. This is also the reason why the resulting 3D reconstructions are less accurate and exhibit more losses near the object’s outline.

Given that the 3D reconstruction of fishes provides the capability of extracting geometric measurements, such as the total length and the height of fishes, which are valuable in the field of Aquaculture, the evaluation of these important measurements related to fishes is essential. Utilizing the 3D reconstructions shown in Figure 17, the total length and height of the fishes, as measured after these 3D reconstructions, as well as in reality, are presented in Table 7.

These results indicate that the proposed method can be utilized for a reliable estimation of the significant geometric measurements of the fish body surfaces, which constitutes a highly valuable advantage of the approach. Furthermore, these estimations are performed without any physical contact between the fishes and humans, but remotely. Finally, the proposed methodology is fully unsupervised and does not carry the drawbacks of many contemporary techniques relying on Deep Learning (such as the need for a training set, specific hardware requirements, etc.).

In the remainder of this section, the presented methodology choices are compared to the respective choices made in other significant works utilizing the CSL technique. Furthermore, the selection of the CSL technique over other reconstruction techniques is justified. In [9], Otsu’s technique is used as a binarization method, which is a global binarization method. Such a technique is not suitable for cases where the 3D object is unevenly illuminated. In our case, the binarization results using Otsu’s method are shown in Figure 20. It is clear that using these binarized images, the performance of the corresponding 3D reconstructions would be poor. Our paper utilizes Sauvola’s adaptive binarization method, which does not derive a single threshold for the entire image but instead operates on small regions of it. In [10], symbol classification is based on geometric rules specifically adapted on the symbols used. In particular, the ring is recognized as the only symbol among the three used, which consists of two concentric circles with very little radius difference. Additionally, the orientation of another type of used symbols, the dashes, plays a significant role in extracting codewords. It is evident that these rules are particularly vulnerable to potential irregularities in the captured surface, which would introduce noise into the image captured by the camera. In our paper, a classification rule is introduced, which is independent of the particular symbols used. Furthermore, the algorithm for extracting codewords does not rely on any specific symbol among those used for the construction of the pattern, but is more general, based on a sequence of extracting the nearest symbols. Other papers, such as those in [29,30,31,32,33], use different colors as a differentiating criterion, and the corresponding patterns are not binary. Another highly significant category of one-shot patterns is those based on De Bruijn encoding [34,35,36]. These are 1D variations of the aforementioned patterns, with a 2D encoding. The typical patterns belonging to this category are also based on different colors. In these cases, the generalization capabilities are significantly limited and are determined by the color of the surface where the pattern is projected. This is also a fundamental reason why we chose a binary pattern, consisting of white geometric symbols placed on a black background.

Multi-shot techniques are generally more efficient than one-shot techniques; however, they are not suitable for scenarios involving moving objects. In our case, the analysis pertains to the 3D reconstruction of fishes, which, in real-world conditions are a part of dynamic scenes. As this work will expand in the future to include the 3D reconstruction of swimming fishes, a one-shot approach—such as the one proposed—is more suitable than a multi-shot technique. Concerning other potential reconstruction techniques, our method utilizes a single-camera setup in conjunction with a projector, effectively acting as a stereo pair. However, the addition of an extra camera can improve both measurement accuracy and the quantity of our measurements by reducing instances of occlusion. Nevertheless, adopting a stereo setup with dual cameras would significantly increase both the cost and the bulkiness of the device. Considering that our proposed system is designed to evaluate the minimal hardware requirements for acquiring adequate data to determine the size of live fish under challenging underwater conditions, this approach is not pursued.

Active stereo systems, comprising two cameras and one projector, are commonly employed in popular RGBD sensors such as Intel RealSense cameras [37]. The primary purpose of incorporating a projector into these systems is to introduce additional details into featureless or challenging areas of the scene. This augmentation provides essential visual cues to the stereo system, consequently leading to a substantial enhancement in the density of 3D measurements. Furthermore, in [38], an active stereo setup employing one projector and two cameras is utilized to enhance the accuracy and robustness of 3D measurements in dynamic and challenging scenes. Similarly, the Structure From Motion (SFM) methodology relies on the identification of significant features within captured images for depth reconstruction. In cases where challenging conditions prevail, such as the absence of image features or complicated subject materials that exhibit variations in color and light intensity from different viewing angles, the resulting 3D data may be incomplete and noisy. Employing a technique akin to active stereo SFM, which introduces features into the scene using a light projector, has been shown to significantly increase the density and accuracy of 3D reconstruction, as demonstrated in [39]. However, adopting SFM for the precise reconstruction of dynamic scenes would necessitate a substantial number of synchronized cameras strategically placed on a specialized rig. This would consequently lead to a considerable increase in both the cost and the physical size of the 3D scanning system.

Finally, it should be noted that the main goal of the structured light 3D scanning system presented in this paper is the one-shot 3D scanning of dynamic scenes, featuring fast moving subjects, such as living fishes. Reconstructing fast moving subjects, for example, using stripes, instead of the proposed single-shot 3D scanning pattern, requires multiple pictures of light stripes with alternating phases and frequencies that cannot be performed instantly. Furthermore, a 3D scanning approach with stripes would naturally increase the acquired 3D measurements from our scene (sampling); however, the accuracy of those measurements would be the same since they are both tracked down to the projected pixel.

## 5. Conclusions

In this paper, a new methodology for the 3D reconstruction of various fish species with a fusiform body shape based on the CSL technique is presented. A new binary pattern is generated to establish correspondences between the pattern and the images captured by the camera. As the problem is investigated specifically in the case of the fish body surfaces, the individual steps of the proposed methodology are adapted based on this context. Specifically, a classification technique is introduced for the symbols, taking into account only the fundamental geometric transformations of rotation and scaling, instead of the more general projective transformation. The classification results as well as the results of the codeword extraction process demonstrate that for the surface of the body of various fish species, which generally satisfy the condition of local smoothness, the corresponding algorithms are quite effective.

Fusiform fishes encompass a wide range of fish species, exhibiting variations in various characteristics. Maximizing the generalization capability of the methodology across multiple categories of fusiform fishes was a factor that influenced the various choices of the proposed methodology. Specifically, some of these challenges and the proposed solutions for addressing them are as follows:

- These fishes might exhibit varying colors. For this reason, a binary pattern was chosen.
- Being living organisms, fishes are mobile. Thus, the proposed pattern is one-shot, suitable for this specific application.
- The bodies of many fusiform fishes are quite large and exhibit various color variations, causing the camera to capture images where the fish bodies are illuminated by the pattern in a non-uniform manner. For this reason, an adaptive binarization method—specifically Sauvola’s method—is employed for an effective image binarization of the images captured by the camera.
- There are regions on the fishes, particularly at the boundaries of their bodies, where curvature changes abruptly. This creates challenges, especially in accurately extracting subpatterns and codewords. To address this, a rather conservative algorithm for subpattern extraction was employed, relying solely on the extraction of adjacent symbols for each symbol.
- Fishes have different areas on their bodies, such as fins, eyes, etc., where corresponding symbols are significantly distorted and do not readily conform to general classification rules. Similarly, as is the case with every 3D object reconstructed using the CSL method, the symbols at the boundaries of the objects may be truncated. In order to address the issue of the potentially erroneous classifications of these symbols, a pattern with the capability to correct one error per codeword was designed.

The proposed methodology for 3D reconstructions of fishes generally results to highly accurate representations of the fishes, as shown in Figure 17, despite some symbol losses, particularly in the contour of the objects. Moreover, these 3D reconstructions closely approximate the actual dimensions of the fishes. One major advantage of the proposed methodology is that it can be accomplished remotely, without requiring physical contact between a person and the fishes, something which is desirable in the field of Aquaculture.

The pattern created in the context of this paper is a one-shot pattern, making it suitable for reconstructing dynamic scenes, where the objects to be reconstructed are in motion. As future work, the proposed methodology is intended to be used in real-life conditions, where the fishes are not in a dry state, as in this paper, but rather swim freely, and the medium through which the projected light propagates will be water instead of air.

## Figures and Tables

**Table 1:** SNR (in dB) of the images captured by the camera for each of the four fish cases used in the experiments.

Fish Type	SNR (in dB)
white seabream	13.68
red seabream	15.90
gilthead seabream	14.62
European seabass	13.10

**Table 2:** The number of symbols contained in the binarized images, as well as the number of valid codewords contained in these images and the final number of corresponding points for the case of white seabream.

Number of symbols in the image, $I_{c}$	380
Number of valid codewords	248
Final number of corresponding points between the pattern and the image, $I_{c}$	362

**Table 3:** The number of symbols contained in the binarized images, as well as the number of valid codewords contained in these images and the final number of corresponding points for the case of red seabream.

Number of symbols in the image, $I_{c}$	275
Number of valid codewords	165
Final number of corresponding points between the pattern and the image, $I_{c}$	254

**Table 4:** The number of symbols contained in the binarized images, as well as the number of valid codewords contained in these images and the final number of corresponding points for the case of gilthead seabream.

Number of symbols in the image, $I_{c}$	437
Number of valid codewords	260
Final number of corresponding points between the pattern and the image, $I_{c}$	387

**Table 5:** The number of symbols contained in the binarized images, as well as the number of valid codewords contained in these images and the final number of corresponding points for the case of European seabass.

Number of symbols in the image, $I_{c}$	330
Number of valid codewords	189
Final number of corresponding points between the pattern and the image, $I_{c}$	320

**Table 6:** The normalized SAD matrix, which contains the normalized SAD of each symbol from each other.

	Symbol	Rectangle	‘L’	‘T’	Triangle	‘X’	‘Π’
Symbol							
Rectangle		0	1.31	1.31	0.97	1.11	1.17
‘L’		1.31	0	1.50	0.89	1.17	0.93
‘T’		1.31	1.50	0	0.98	1.03	1.06
Triangle		0.97	0.89	0.98	0	0.73	1.47
‘X’		1.11	1.17	1.03	0.73	0	1.29
‘Π’		1.17	0.93	1.06	1.47	1.29	0

**Table 7:** The actual total length and height as well as the experimentally evaluated measurements obtained for the four fishes used in our experiments. All measurements are in cm.

	Fish	White Seabream	Red Seabream	Gilthead Seabream	European Seabass
Measurement					
Total length resulting from 3D reconstruction		29.8	27.5	32.7	31.2
Actual total length		30.6	26.0	31.7	31.7
Height resulting from 3D reconstruction		11.2	11.0	10.9	7.4
Actual height		11.2	10.4	11.0	7.8

## Data Availability

Not applicable.
